# Development and validation of risk of CPS decline (RCD): a new prediction tool for worsening cognitive performance among home care clients in Canada

**DOI:** 10.1186/s12877-023-04463-3

**Published:** 2023-12-01

**Authors:** Dawn M. Guthrie, Nicole Williams, Hannah M. O’Rourke, Joseph B. Orange, Natalie Phillips, M. Kathleen Pichora-Fuller, Marie Y. Savundranayagam, Rinku Sutradhar

**Affiliations:** 1 Department of Kinesiology & Physical Education, Wilfrid Laurier University, Waterloo, ON Canada; 2 Department of Health Sciences, Wilfrid Laurier University, Waterloo, ON Canada; 3 College of Health Sciences, Faculty of Nursing, University of Alberta, Edmonton, AB Canada; 4 School of Communication Sciences and Disorders, Western University, London, ON Canada; 5 Department of Psychology, Centre for Research in Human Development, Concordia University, Montreal, QC Canada; 6 Department of Psychology, University of Toronto, Mississauga, ON Canada; 7 School of Health Studies, Western University, London, ON Canada; 8 Institute of Health Policy, Management and Evaluation, University of Toronto, Toronto, ON Canada

**Keywords:** Prediction tool, interRAI, Home care, Cognitive Performance Scale, Standardized assessment, Decision support

## Abstract

**Background:**

To develop and validate a prediction tool, or nomogram, for the risk of a decline in cognitive performance based on the interRAI Cognitive Performance Scale (CPS).

**Methods:**

Retrospective, population-based, cohort study using Canadian Resident Assessment Instrument for Home Care (RAI-HC) data, collected between 2010 and 2018. Eligible home care clients, aged 18+, with at least two assessments were selected randomly for model derivation (75%) and validation (25%). All clients had a CPS score of zero (intact) or one (borderline intact) on intake into the home care program, out of a possible score of six. All individuals had to remain as home care recipients for the six months observation window in order to be included in the analysis. The primary outcome was any degree of worsening (i.e., increase) on the CPS score within six months. Using the derivation cohort, we developed a multivariable logistic regression model to predict the risk of a deterioration in the CPS score. Model performance was assessed on the validation cohort using discrimination and calibration plots.

**Results:**

We identified 39,292 eligible home care clients, with a median age of 79.0 years, 62.3% were female, 38.8% were married and 38.6% lived alone. On average, 30.3% experienced a worsening on the CPS score within the six-month window (i.e., a change from 0 or 1 to 2, 3, 4, 5, or 6). The final model had good discrimination (c-statistic of 0.65), with excellent calibration.

**Conclusions:**

The model accurately predicted the risk of deterioration on the CPS score over six months among home care clients. This type of predictive model may provide useful information to support decisions for home care clinicians who use interRAI data internationally.

## Background

The World Health Organization estimates that 55 million people globally have dementia, with the number expected to rise to 78 million within the next decade [1]. They also note that dementia tends to be under-diagnosed and the diagnosis often comes relatively late in the person’s disease trajectory [2]. Early detection of changes to a person’s cognitive performance (i.e., changes to a person’s function that are indicators of and associated with cognitive impairment) that may suggest cognitive impairment (CI) is key for several reasons. Early detection of cognitive impairment enables clinicians to identify and treat some modifiable contributors to cognitive changes, such as delirium, increased pressure or bleeding in the brain, vitamin deficiencies, or depression [3]. CI is a known risk factor for several negative outcomes including caregiver burden or distress [4, 5], repeat visits to the emergency department [6], and admission to a long-term care (LTC) facility or nursing home [7–11]. It also is important for clinicians to identify and to track changes to cognitive performance over time, because of its association with CI, and since some evidence suggests that mild CI is an intermediate step in the development of dementia [12, 13]. Furthermore, despite the lack of disease altering treatments for dementia, it is important to identify its onset and to provide symptom altering treatment options as early as possible [14]. Early detection of people who may be at risk for cognitive decline is critical therefore to enable timely intervention, which could delay disease development or progression [15]. Information about risks for decline in cognitive performance could be used to identify non-pharmacological approaches to address critical risk factors for decline such as diet, physical inactivity, obesity, hearing loss, and social isolation [16], among those who are and are not diagnosed with a disease that causes CI.

A simple but robust method is needed to assist clinicians in identifying individuals who are at risk of a deterioration in their cognitive performance. Similar types of risk prediction tools, or nomograms, have been used widely in cancer care [17–19]. However, very few exist for predicting changes in cognitive performance [20]. Unlike multivariable regression models that typically focus on exposure-outcome effect estimates, nomograms focus on estimating an individual’s predicted outcome probability based on their specific profile of characteristics.

Understanding the risk of deterioration in cognitive performance is particularly important within the home care sector. Roughly two million Canadians receive publicly-funded home care annually, and around 40% of them are aged 65 + [21]. It is also recognized that home care clients are generally more impaired in their cognitive functioning as compared to other older adults not receiving this type of care. Studies in home care report rates of CI ranging from 30% in Europe [22, 23], 27%-38% in Australia [24, 25], and 40%-60%in Canada [26–30], as compared to roughly 3% among, community-dwelling older persons not receiving home care [31].

The interRAI Cognitive Performance Scale (CPS) [32] was designed to be a functional measure and to act as a brief screen for impaired cognitive performance. Among Canadian home care recipients, roughly 20% would be expected to experience a decline in their CPS score over a one-year period [26]. Although it is not a diagnostic tool, some data have shown that the proportion of individuals diagnosed with dementia increases with each 1-point increase (i.e., worsening) on the CPS score [33]. Furthermore, a one-point change on the lower values of the CPS (e.g., those scoring a zero or one on the CPS) corresponds to a roughly 2.4- to 3.0-point difference on the Mini-Mental State Examination (MMSE) [32, 34]. These values exceed the 1.4-point change on the MMSE, suggested by Howard et al. [35], to represent the minimal clinically important difference. In a cognitively healthy cohort, changes on the MMSE over three months were roughly 0.35 to 0.69 [36]. All of this supports the use of the CPS in detecting a clinically relevant change over time.

The CPS is a hierarchical scale that includes ratings of two domains found on traditional cognitive assessments (e.g., difficulties in short-term memory, daily decision making) and two items reflecting functional status (e.g., expressive communication, independence in eating). The scale ranges from zero to six (0 = no impairment in cognitive functioning, 1 = borderline intact, 2 = mild impairment, 3 = moderate impairment, 4 = moderately severe impairment, 5 = severe impairment, and 6 = very severe impairment in cognitive functioning). The CPS is embedded within multiple clinical assessment tools developed by interRAI, a non-profit consortium of researchers, clinicians, and policy makers from roughly 37 countries. The items within the CPS have excellent inter-rater reliability within the LTC population (average kappa = 0.85, which measures the extent to which assessors assign the same score) [32] and good reliability in home care (average kappa = 0.65) [37]. In multiple studies, the CPS has demonstrated at least moderate correlation (values of 0.45 and higher) with performance on two cognitive screening measures, namely the Mini-Mental State Examination [32, 34, 38–42] and the Montreal Cognitive Assessment [43].

In this paper, we aimed to develop and validate a new nomogram, the risk of CPS decline (RCD). Specifically, this tool was created to estimate the predicted 6-month risk of a decline on the CPS among individuals with a baseline CPS score of zero or one. The long-term goal of this work would be to have the predicted probability, for an individual client, included in the outputs available to clinicians, similar to the other scales and algorithms that can be generated with the interRAI Home Care assessment. As part of the validation process, another objective was to explore the characteristics of clients in the highest risk versus the lowest risk groups. During our preliminary analysis, it was clear that individuals with a baseline CPS score of two or higher had a different risk profile, and warranted a unique nomogram. Those results will be reported in a separate manuscript.

## Methods

### Data source

We conducted secondary analysis of data collected using the Resident Assessment Instrument for Home Care (RAI-HC) across five provinces (British Columbia, Alberta, Manitoba, Ontario, Newfoundland and Labrador) and one territory (Yukon Territory) in Canada. The development and validation of the prediction tool took place between Nov. 2020 and July 2022. The RAI-HC is a standardized assessment that is used routinely for all home care clients expected to receive at least 60 days of service [44]. The assessment has established reliability and validity and contains roughly 300 items which capture key domains, including cognitive functioning, sensory impairments and functional ability [37]. Assessments are completed by trained care coordinators (typically registered nurses) through discussion with the individual, their informal care providers and other health care professionals, as needed. Re-assessments are typically completed every 6–12 months or following a change in health status [45]. Missing data are rare as the electronic assessment does not allow assessors to close an assessment when fields are left blank.

At the time of the analysis, two different interRAI home care instruments were available for use, namely, the RAI-HC and also the newer version of this instrument, the interRAI Home Care Assessment (or interRAI HC). These two assessments are very similar, but there are roughly 100 items within the interRAI HC which are not found on the RAI-HC assessment. Our goal was to create a nomogram that would be compatible with either version of the instrument. As a result, we excluded from consideration any item that was not available on the interRAI HC instrument. We opted to analyze existing RAI-HC data across several jurisdictions in Canada since this yielded the largest database available. At the time, very limited interRAI HC data were available, and only for the province of Ontario. The Research and Ethics Board at Wilfrid Laurier University reviewed and approved the design of this study (#6504).

### Sample

The sample included all home care clients who were 18 years of age or older and who had at least two RAI-HC assessments completed between January 2010 and December 2018. Only individuals whose first assessment was an intake assessment were retained, and each individual had to have at least one additional assessment completed within six months following their baseline (intake) assessment. For the vast majority of clients (76.9%), the reason for the re-assessment was either a regular follow-up assessment or a routine assessment at a fixed interval. Home care clients had to be receiving home care throughout the entire duration of the six-month period to be included. Since the main objective of the current study was to develop a nomogram predicting any decline on the CPS (vs. no decline) within six months for individuals with a baseline CPS score of zero or one, only those individuals were included in the sample (*n* = 39,292). The choice to define our main outcome as dichotomous (any decline vs. no decline on the CPS) was an *a prior*i clinical decision, not a statistical one.

### Covariates

All characteristics were measured once at baseline. They included: demographic characteristics (age at intake, sex [male vs. female], marital status, caregiver relationship to client, disease diagnoses [stroke, congestive heart failure, coronary artery disease, Alzheimer’s dementia, another type of dementia, hemiplegia/hemiparesis, multiple sclerosis, Parkinson’s disease, any psychiatric diagnosis, hip fracture, other types of fractures, pneumonia, urinary tract infection, cancer, diabetes, chronic obstructive pulmonary disease], sudden/new onset change in mental function [each coded as yes or no]), sensory and communication challenges (hearing impairment [HI], vision impairment [VI], dual sensory impairment [DSI; yes or no], the ability to understand others), health conditions and responsive behaviours [each yes or no] (wandering, verbally abusive, physically abusive, socially inappropriate, resists care, chest pain, no bowel movements, dizziness or light-headedness, edema, shortness of breath, delusions, hallucinations, smoked/chewed tobacco).

Additionally, items around physical functioning and health status (client believes they are capable of increased functional independence [yes or no], number of falls [0, 1 or 2 +], unsteady gait, bladder incontinence, client believes they have poor health, client has condition or diseases that make cognition, activities of daily living [ADL]), mood or behaviour patterns unstable, flare-up of recurrent or chronic condition, prognosis of less than six months to live, difficulty swallowing, ate one or fewer meals in the last three days, unintended weight loss [each yes or no]), service utilization (hospital admissions, emergency department visits [both 0, 1, or 2 +], made economic trade-offs during the last month [yes or no] and social functioning [client indicates that they feel lonely, change in social activities causing distress; code as yes or no]) were all explored in the model.

Finally, seven health index scales/algorithms embedded within the RAI-HC which are automatically generated upon completion of the assessment also were explored. Across all scales, a higher value indicates a great degree of impairment.

1. *The Activities of Daily Living (ADL) Self-Performance Hierarchy Scale* includes four items, namely, bathing, dressing, toilet use, locomotion, and eating. It is scored from zero (no difficulty) to six (major difficulty), where a cut-point of two or higher was used to indicate at least moderate difficulty completing ADLs independently [46].
2. *The Instrumental Activities of Daily Living (IADL) Involvement Scale* is a summative scale across seven IADLs (meal preparation, housework, managing finances, managing medications, phone use, shopping, and transportation), which ranges from 0 to 21, where a cut-point of 14 or higher was used to indicate moderate difficulty completing these tasks. Both the ADL and IADL scales are valid and reliable measures of functional ability [46].
3. *The Depression Rating Scale (DRS)* includes seven items related to mood and behaviour. The scale ranges from 0 to 14 where a score of three or higher is predictive of a clinical diagnosis of depression [47].
4. *The Pain Scale* uses two items, one related to pain frequency and one related to intensity. It is measured from zero (no pain/less than daily pain) to four (daily/severe pain) and a cut-point of two or higher was used to indicate pain that was daily or severe. The scale has been validated against the vertical version of the Visual Analog Scale [48].
5. *The Changes in Health, End-Stage Disease Signs and Symptoms (CHESS) Scale* uses nine items including shortness of breath, vomiting, dehydration, and prognosis. It can range from zero to five. For every one-point increase on the scale, there is a nearly two-fold increased risk of mortality [49].
6. *The Pressure Ulcer Risk Scale (PURS)* is scored from zero to eight and groups clients into low, moderate, high, and very high risk of experiencing a pressure ulcer. It includes seven items such as bowel incontinence, weight loss, history of a resolved pressure ulcer, and impaired bed mobility [50].
7. *The Caregiver Risk Evaluation (CaRE)* is a decision-support tool that generates the risk of caregiver burden among informal caregivers. It contains six individual items or scores on the health index scales (e.g., CPS scale and DRS scale) and assigns caregivers into one of four groups, ranging from low risk (score of zero) to very high risk (score of four) of experiencing burden [7].

### Analysis

#### Developing the prediction model

We randomly selected 75% of eligible home care clients for model derivation and used the remaining 25% for validation. To check how well random sampling produced equivalent groups, we compared the distributions of baseline characteristics between the derivation and validation cohorts. Using the derivation cohort, we used a multivariable logistic regression model to predict a decline on the CPS score within 6 months of an individual’s baseline assessment. Numerous variable selection techniques were initially explored for deriving a parsimonious model (e.g., backward, forward, and stepwise procedures), and these techniques showed consistent preliminary results. Similar to prior work, our final model focused on the backward selection procedure for variable selection [18, 19]. We chose a more liberal two-tailed alpha value of 0.10, to ensure that important interaction terms would not be neglected. In addition to the p-value, we examined the AIC and log-likelihood values from our series of models during the iterative model building process. Our final model had the lowest AIC and highest log-likelihood, while retaining main effects and interactions that were clinically meaningful. Continuous variables such as age were explored using both linear and quadratic terms. Missing data were only an issue when using the CaRE algorithm to categorize the risk of caregiver burden, as this measure is only calculated when all items in the algorithm are not missing. If an individual did not have a primary caregiver, then the value would be set to missing. Because there was no obvious missing pattern, we created a missing category for these individuals rather than impute or remove them from the analysis. As decided a priori, all two-way interactions with age and sex were explored, along with other two-way interactions with each of the three types of sensory impairments. For example, we explored two-way interactions with each of HI, VI and DSI with all of the following covariates: Alzheimer’s dementia or another type of dementia, Parkinson’s disease, any psychiatric diagnosis, sudden/new onset change in mental function, the CaRE algorithm, number of falls and ability to understand others.

#### Validating the prediction model

Once the optimal regression model for predicting CPS decline was developed using the derivation cohort, the validation cohort then was used to assess the performance of this model. Specifically, for each individual in the validation cohort, the estimated predicted probability of decline on the CPS score within 6 months was calculated based on their specific baseline covariate values and the corresponding regression coefficient (beta) estimates from the regression model. Calibration was examined by grouping individuals into deciles (or groups) of lowest to highest risk and then plotting the observed proportion of CPS decline within a decile against the corresponding mean predicted risk within that decile. Points closer to the 45-degree line indicate better calibration [18]. We examined the calibration plot overall, as well as examined the plots within various sub-groups (e.g., sex, baseline CPS score, HI, presence of any type of dementia, number of falls [0 vs. 1 +], ability to understand others [any difficulty vs. none]) in order to assess whether model calibration was different within these groups. The model’s discriminative ability (i.e., ability to discriminate between those who declined from those who did not decline) was measured via the area under the curve (AUC) statistic, where a value of 1.0 implies perfect discrimination and a value of 0.5 implies the model classifies no better than chance [18, 19]. All analyses were performed using SAS software, version 9.4 [51]. This study followed the Transparent Reporting of a Multivariable Prediction Model for Individual Prognosis or Diagnosis reporting guidelines [52].

## Results

A total of 39,292 individuals had a baseline CPS score of zero or one between 2010 and 2018. The median age of the overall sample was 79.0 years (interquartile range = 62–96 years), 62.2% were female, 38.8% were married and 38.6% lived alone. Distributions of characteristics between the derivation and validation cohorts were nearly identical at the intake assessment (Table 1). On average, 30.3% experienced a worsening on the CPS score within 6 months following their baseline assessment, which was nearly identical between males and females (males: 30.9%; females: 30.3%). Most clients experienced a one-point decline (70.2%; from CPS zero to one or from CPS of one to two), 25.9% experienced a two-point decline, and the rest, a three-point worsening on the CPS (3.9%).

**Table 1:** Comparison of the derivation and validation cohorts across all baseline variables under consideration from the RAI-HC

	Derivation cohort (*n* = 29,497)	Validation cohort (*n* = 9,795)	*p*-value
	% (n)		
**Age (years)**			
18–64	18.1 (5,335)	18.5 (1,8810)	0.7131
65–74	17.8 (5,252)	17.4 (1,705)	
75–84	33.3 (9,824)	33.5 (3,276)	
85 +	30.8 (9,086)	30.7 (3,004)	
**Sex**			
Male	37.5 (11,070)	38.2 (3,741)	0.2402
Female	62.5 (18,427)	61.8 (6,054)	
**Marital status**			
Never married	7.7 (2,262)	7.7 (751)	0.9940
Married	38.8 (11,436)	38.7 (3,789)	
Widowed	36.2 (10,666)	36.3 (3,559)	
Separated/divorced	10.5 (3,093)	10.5 (1,030)	
Unknown	6.9 (2,040)	6.8 (666)	
**Who lived with at time of referral**			
Alone	38.6 (11,388)	38.7 (3,791)	0.1707
Spouse only	31.3 (9,243)	31.7 (3,104)	
Spouse and others	8.1 (2,374)	7.4 (728)	
Child	11.2 (3,316)	11.1 (1,085)	
Others (not spouse or child)	6.3 (1,857)	6.3 (616)	
Group setting	3.8 (1,126)	3.9 (386)	
Missing	0.7 (193)	0.9 (85)	
**Province/territory**			
Ontario	76.6 (22,607)	75.7 (7,419)	0.0216
British Columbia	13.3 (3,390)	13.5 (1,3200	
Alberta	4.0 (1,189)	4.1 (403)	
Manitoba	3.5 (1,025)	3.7 (364)	
Newfoundland and Labrador	2.5 (741)	2.9 (282)	
Yukon Territory	0.02 (5)	0.01 (7)	
**Year of intake assessment**			
2010	17.5 (5,177)	17.7 (1,738)	0.1738
2011	15.9 (4,685)	15.2 (1,484)	
2012	12.7 (3,754)	12.7 (1,241)	
2013	10.1 (2,979)	10.2 (1,000)	
2014	11.0 (3,243)	10.7 (1,052)	
2015	11.8 (3,475)	12.8 (1,251)	
2016	11.7 (3,440)	11.3 (11,05)	
2017	9.3 (2,744)	9.4 (924)	
**Baseline CPS score**			
0	60.8 (17,938)	61.3 (6,003)	0.4054
1	39.2 (11,559)	38.7 (3,792)	
**Health Index Scales**			
**Activities of Daily Living (ADL) Self-Performance Hierarchy Scale**			
None/minor difficulty (0–1)	74.4 (21,956)	74.7 (7,314)	0.6424
Moderate/major difficulty (2–6)	25.6 (7,541)	25.3 (2,481)	
**Instrumental Activities of Daily Living (IADL) Involvement Scale**			
None/minor difficulty (0–13)	71.5 (21,085)	72.1 (7,066)	0.2113
Moderate/major difficulty (14–21)	28.5 (8,412)	27.9 (2,729)	
**Depression Rating Scale (DRS)**			
No signs/symptoms (0–2)	81.0 (23,881)	80.2 (7,851)	0.0789
Signs/symptoms of depression (3–14)	19.0 (5,616)	19.8 (1,944)	
**Pain Scale**			
No pain/less than daily pain (0–2)	81.2 (23,957)	80.9 (7,925)	0.4971
Severe/daily pain (3–4)	18.8 (5,540)	19.1 (18,70)	
**Change in Health, End-stage disease Signs and Symptoms Scale (CHESS)**			
None/mild health instability (0–1)	52.9 (15,617)	53.0 (5,189)	0.9566
Moderate/severe health instability (2–5)	47.1 (13,880)	47.0 (4,606)	
**Pressure Ulcer Risk Scale (PURS)**			
Low risk	86.9 (25,642)	86.6 (8,482)	0.6951
Moderate risk	8.8 (2,590)	9.1 (893)	
High risk	4.0 (1,188)	4.1 (398)	
Very high risk	0.3 (77)	0.2 (22)	
**Caregiver status**			
** Caregiver lives with client**			
No	46.8 (13,800)	46.7 (4,575)	0.9235
Yes	49.6 (14,642)	49.8 (4,877)	
No caregiver	3.6 (1,055)	3.5 (343)	
** Caregiver relationship to client**			
Child	46.5 (13,715)	46.6 (4,562)	0.9293
Spouse	30.7 (9,058)	30.9 (3,025)	
Other relative/friend/neighbor	18.9 (5,576)	18.8 (1,839)	
No caregiver	3.6 (1,055)	3.5 (343)	
Missing	0.3 (93)	0.3 (26)	
** Caregiver Risk Evaluation (CaRE)**			
Low risk	25.4 (7,497)	256.2 (2,568)	0.4534
Moderate risk	33.3 (9,832)	33.1 (3,241)	
High risk	37.3 (10,991)	36.8 (3,607)	
Very high risk^a^	n/a	n/a	
Missing	4.0 (1,177)	3.9 (379)	
** Client openly expresses conflict or anger with family/friends**			
No	86.1 (25,401)	86.0 (8,421)	0.7261
Yes	13.9 (4,096)	14.0 (1,374)	
**Disease diagnoses (reference = not present)**			
Stroke	12.7 (3,740)	13.0 (1,269)	0.4774
Congestive heart failure	12.0 (3,546)	12.4 (1,217)	0.2895
Coronary artery disease	21.1 (6,215)	20.5 (2,010)	0.2470
Alzheimer’s dementia	0.9 (272)	0.8 (74)	0.1261
Dementia (not Alzheimer’s dementia)	3.4 (998)	3.1 (301)	0.1366
Hemiplegia/hemiparesis	2.2 (641)	2.2 (217)	0.8039
Multiple sclerosis	1.4 (426)	1.4 (139)	0.8564
Parkinson’s disease	4.3 (1,261)	4.3 (421)	0.9220
Any psychiatric diagnosis	14.1 (4,171)	14.5 (1,423)	0.3418
Hip fracture	3.9 (1,152)	4.0 (395)	0.5749
Other fracture	8.9 (2,614)	8.8 (866)	0.9502
Pneumonia	3.0 (895)	3.4 (328)	0.1205
UTI	5.3 (1,551)	5.4 (529)	0.5851
Cancer	16.9 (4,981)	17.0 (1,667)	0.7620
Diabetes	26.9 (7,939)	26.8 (2,622)	0.7779
Emphysema/COPD/asthma	19.4 (5,718)	19.8 (1,941)	0.3506
Sudden/new onset change in mental function (last 7 days)	0.9 (250)	0.8)74)	0.3827
**Communication and sensory impairments**			
**Hearing impairment**			
No hearing impairment	76.4 (22,523)	77.1 (7,555)	0.1171
Hearing impairment only	23.6 (6,974)	22.9 (2,240)	
** Vision impairment**			
No vision impairment	87.5 (25,803)	87.7 (8,587)	0.6211
Vision impairment only	12.5 (3.694)	12.3 (1,208)	
** Dual sensory impairment**			
No dual sensory impairment	89.4 (26,371)	89.6 (8,778)	0.5486
Dual sensory impairment	10.6 (3,126)	10.4 (1,017)	
** Ability to understand others**			
Understands	91.1 (26,884)	90.8 (8,905)	0.4083
Usually understands	7.9 (2,343)	8.2 (806)	
Often understands	0.8 (235)	0.8 (77)	
Sometimes understands	0.1 (32)	0.1 (5)	
Rarely/never understands	0.01 (3)	0.02 (2)	
**Health conditions and behaviours**			
**Wandering**			
No	99.9 (29,455)	99.9 (9,784)	0.4821
Yes	0.1 (42)	0.1 (11)	
** Verbally abusive**			
No	98.9 (29,165)	98.8 (9,676)	0.4719
Yes	1.1 (332)	1.2 (119)	
** Physically abusive**			
No	99.9 (29,462)	99.9 (9,782)	0.7299
Yes	0.1 (35)	0.1 (13)	
** Socially inappropriate**			
No	99.6 (29,388)	99.7 (9,762)	0.6411
Yes	0.4 (109)	0.3 (33)	
** Resists care**			
No	98.6 (29,071)	98.3 (9,632)	0.1207
Yes	1.4 (426)	1.7 (163)	
** Chest pain**			
No	94.9 (27,980)	95.3 (9,334)	0.0870
Yes	5.1 (1,517)	4.7 (461)	
** No bowel movement in last 3 days**			
No	97.8 (11,308)	97.9 (17,557)	0.7834
Yes	2.2 (251)	2.1 (381)	
** Dizziness or light-headedness**			
No	75.6 (8,733)	80.0 (14,345)	< 0.0001
Yes	24.4 (2,826)	20.0 (3,593)	
** Edema**			
No	68.0 (20,051)	67.6 (6,623)	0.5082
Yes	32.0 (9,446)	32.4 (3,172)	
** Shortness of breath**			
No	69.1 (20,385)	68.9 (6,751)	0.7303
Yes	30.9 (9,112)	31.1 (3,044)	
** Delusions**			
No	99.6 (29,375)	99.5 (9,745)	0.2083
Yes	0.4 (122)	0.5 (50)	
** Hallucinations**			
No	99.1 (29,234)	99.3 (9,729)	0.0404
Yes	0.9 (263)	0.7 (66)	
** Smoked/chewed tobacco daily**			
No	90.4 (26,668)	90.5 (8,866)	0.7564
Yes	9.6 (2,829)	9.5 (929)	
**Physical functioning and health status**			
** Client believes they are capable of increased functional independence**			
No	68.4 (20,170)	68.8 (6,739)	0.4376
Yes	31.6 (9,327)	31.2 (3,056)	
** Number of days in the last 7 days client went out of the house**			
Every day	13.5 (3,980)	13.7 (1,344)	0.7713
2–6 days a week	34.3 (10,103)	33.8 (3,314)	
1 day a week	34.2 (10,087)	34.6 (3,387)	
No days	18.1 (5,327)	17.9 (1,750)	
** Number of falls in last 90 days**			
No falls	56.8 (16,750)	57.0 (5,585)	0.8531
1 fall	22.9 (6,765)	23.0 (2,249)	
2 or more falls	20.3 (5,982)	20.0 (1,961)	
** Unsteady gait**			
No	31.2 (9,199)	31.7 (3,105)	0.3423
Yes	68.8 (20,298)	68.3 (6,690)	
** Client limits going outdoors due to fear of falling**			
No	51.7 (15,250)	52.2 (5,116)	0.3625
Yes	48.3 (14,247)	47.8 (4,679)	
** Bladder incontinence in last 7 days**			
Continent	59.3 (17,480)	59.8 (5,856)	0.3590
Incontinent	40.7 (12,017)	40.2 (3,939)	
** Client believes he/she has poor health**			
No	72.9 (21,502)	73.1 (7,163)	0.6520
Yes	27.1 (7,995)	26.9 (2,632)	
** Client has conditions or diseases that make cognition, ADL, mood or behavior patterns unstable**			
No	64.5 (19,034)	64.1 (6,278)	0.4362
Yes	35.5 (10,463)	35.9 (3,517)	
** Client experienced a flare-up of a recurrent/chronic problem**			
No	84.7 (24,978)	84.4 (8,270)	0.5540
Yes	15.3 (4,519)	15.6 (1,525)	
** Client has a prognosis of less than six months to live**			
No	98.5 (29,051)	98.4 (9,640)	0.622
Yes	1.5 (446)	1.6 (155)	
** Number of medications**			
0–4	18.3 (5,393)	18.5 (1,808)	0.6978
5 +	81.7 (24,104)	81.5 (7,987)	
** Swallowing**			
Normal	93.0 (274,435)	92.8 (9,091)	0.5095
Requires modifications to swallow (e.g., diet, tube feeding, etc.)	7.0 (2,062)	7.2 (704)	
** Ate one or fewer meals a day in last 3 days**			
No	95.5 (28,167)	95.6 (9,365)	0.6220
Yes	4.5 (1,330)	4.4 (430)	
** Unintended weight loss of 5% or more in last 30 days**			
No	87.3 (25,756)	87.1 (8,535)	0.6142
Yes	12.7 (3,741)	12.9 (1,260)	
**Service utilization**			
**Hospital admissions (last 90 days)**			
0	54.0 (15,913)	53.8 (5,268)	0.8942
1	38.5 (11,358)	38.8 (3,796)	
2 or more	7.5 (2,226)	7.5 (731)	
** Emergency department visits (last 90 days)**			
0	72.8 (21,459)	72.8 (7,132)	0.7998
1	19.2 (5,663)	19.3 (1,894)	
2 or more	8.0 (2,375)	7.9 (769)	
** Made economic trade-offs during the last month**			
No	96.9 (28,591)	97.1 (9,512)	0.3616
Yes	3.1 (906)	2.9 (283)	
**Social functioning**			
**Client indicates that he/she feels lonely**			
No	84.1 (24,812)	83.6 (8,191)	0.2492
Yes	15.9 (4,685)	16.4 (1,604)	
** Change in social activities in last 90 days**			
No decline/decline, not distressed	79.3 (23,377)	78.5 (7,693)	0.1333
Decline, distressed	20.7 (6,120)	21.5 (2,102)	

^a^The very high risk group on the CaRE algorithm is only calculated for individuals with a baseline CPS score of 2 +

After utilizing a backward variable selection approach, a total of 34 main effects and 12 two-way interactions were included in the final risk prediction model. Of these 12 interactions, seven involved sex, including a significant interaction between age and sex. It was seen that the effect of females versus males increased with age. For example, for an individual who was 69 years of age, there was virtually no difference in the odds ratio comparing males and females (OR = 0.99). However, for an individual who was 79, the OR decreased to 0.94, and then decreased again, to 0.90 for someone who was 86.

A number of factors were associated with an increased risk of experiencing a decline on the CPS score of more than 10%, including age (5-year increments), baseline CPS score, IADL impairment, a diagnosis of Alzheimer’s dementia, another type of dementia, Parkinson’s disease and any psychiatric diagnoses, vision impairment, dual sensory impairment, the ability to understand others, wandering, verbally abusive behaviour, hallucinations, the number of falls, and self-reported loneliness (Table 2). The AUC value was 0.6576 in the derivation cohort.

**Table 2:** Estimates from the regression model (main effects only^a^) following backwards elimination for the derivation cohort from the RAI-HC

Parameter	Odds ratio (95% CI)	*p* value
Age (5-year increments)	1.13 (1.12, 1.15)	< 0.0001
**Sex**		
Male	Reference	Reference
Female	0.95 (0.89, 1.00)	0.0067
**Marital status**		
Never married	Reference	Reference
Married	0.85 (0.76, 0.96)	0.0076
Widowed	0.90 (0.80, 1.02)	0.0868
Separated/divorced	1.08 (0.95, 1.22)	0.2744
Other	1.29 (1.12, 1.48)	0.0004
**Baseline CPS score**		
0	Reference	Reference
1	1.21 (1.15, 1.28)	< 0.0001
**Instrumental Activities of Daily Living (IADL) Involvement Scale**		
None/mild difficulty (0–13)	Reference	Reference
Moderate/major difficulty (14–21)	1.17 (1.10, 1.24)	< 0.0001
**Pain Scale**		
No pain/less than daily pain (0–2)	Reference	Reference
Daily/severe pain (3–4)	0.92 (0.86, 0.99)	0.0167
**Caregiver Risk Evaluation (CaRE)**		
Low risk	Reference	Reference
Moderate risk	1.08 (1.00, 1.16)	0.0528
High risk	1.19 (1.10, 1.27)	< 0.0001
Very high risk^a^	n/a	n/a
Missing	1.07 (0.93, 1.23)	0.2824
**Coronary artery disease**		
Not present	Reference	Reference
Present	0.92 (0.86, 0.98)	0.0121
**Alzheimer’s dementia**		
Not present	Reference	Reference
Present	3.84 (2.94, 5.01)	< 0.0001
**Dementia (not Alzheimer’s dementia)**		
Not present	Reference	Reference
Present	2.65 (2.32, 3.04)	< 0.0001
**Parkinson’s disease**		
Not present	Reference	Reference
Present	1.47 (1.30, 1.65)	< 0.0001
**Any psychiatric disorder**		
Not present	Reference	Reference
Present	1.17 (1.08, 1.26)	< 0.0001
**Sudden/new onset change in mental function (last 7 days)**		
No	Reference	Reference
Yes	1.31 (1.00, 1.71)	0.0488
**Stroke**		
Not present	Reference	Reference
Present	1.08 (1.00, 1.17)	0.0426
**Hip fracture**		
Not present	Reference	Reference
Present	0.82 (0.71, 0.94)	0.0334
**Other type of fracture**		
Not present	Reference	Reference
Present	0.91 (0.83, 1.00)	0.0419
**Pneumonia**		
Not present	Reference	Reference
Present	0.82 (0.70, 0.96)	0.0147
**Cancer**		
Not present	Reference	Reference
Present	0.88 (0.81, 0.95)	0.0006
**Hearing impairment**		
No hearing impairment	Reference	Reference
Hearing impairment	1.07 (1.01, 1.15)	0.0352
**Vision impairment**		
No vision impairment	Reference	Reference
Vision impairment	1.14 (1.05, 1.24)	0.0013
**Dual sensory impairment**		
No dual sensory impairment	Reference	Reference
Dual sensory impairment	1.13 (1.03, 1.23)	0.0085
**Ability to understand others**		
Understands	Reference	Reference
Usually understands	1.22 (1.11, 1.33)	< 0.0001
Often understands	1.34 (1.02, 1.76)	0.0336
Sometimes understands	1.32 (0.63, 2.75)	0.4658
Rarely/never understands	2.64 (0.20, 28.0)	0.4953
**Wandering**		
No	Reference	Reference
Yes	4.17 (1.90, 9.16)	0.0004
**Verbally abusive**		
No	Reference	Reference
Yes	1.38 (1.09, 1.74)	0.0080
**Chest pain**		
No	Reference	Reference
Yes	0.84 (0.74, 0.95)	0.0059
**Edema**		
No	Reference	Reference
Yes	0.87 (0.82, 0.92)	< 0.0001
**Hallucinations**		
No	Reference	Reference
Yes	1.41 (1.08, 1.82)	0.0105
**Client believes they are capable of increased functional independence**		
No	Reference	Reference
Yes	0.88 (0.83, 0.94)	< 0.0001
**Number of falls in last 90 days**		
0	Reference	Reference
1	1.13 (1.06, 1.20)	0.0003
2 +	1.32 (1.24, 1.41)	< 0.0001
**Bladder incontinence in last 7 days**		
No	Reference	Reference
Yes	1.09 (1.03, 1.15)	0.0027
**Client has a prognosis of less than 6 months to live**		
No	Reference	Reference
Yes	0.79 (0.62, 1.00)	0.0463
**Number of hospital admissions in last 90 days**		
0	Reference	Reference
1	0.92 (0.87, 0.97)	0.0032
2 +	0.94 (0.85, 1.05)	0.2701
**Client indicates that he/she feels lonely**		
No	Reference	Reference
Yes	1.15 (1.07, 1.23)	0.0002
**Change in social activities in last 90 days**		
No decline/declined, not distressed	Reference	Reference
Declined, distressed	0.91 (0.85, 0.97)	0.0037

^a^ The odds ratio estimates are from the main effects-only model (without the interactions between age, sex, and sensory impairments)

^b^The very high-risk group on the CaRE algorithm is only calculated for individuals with a baseline CPS score of 2 +

**Fig. 1:**
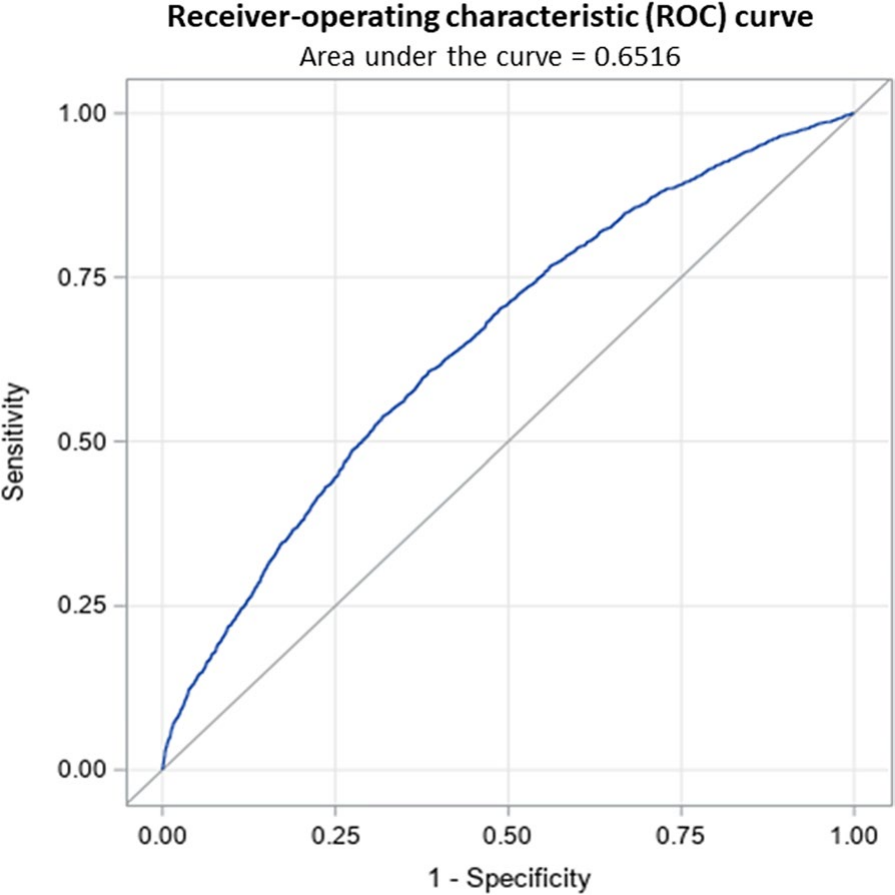
Receiver Operating Characteristic (ROC) curve and the Area Under the Curve (AUC) for the validation cohort

**Fig. 2:**
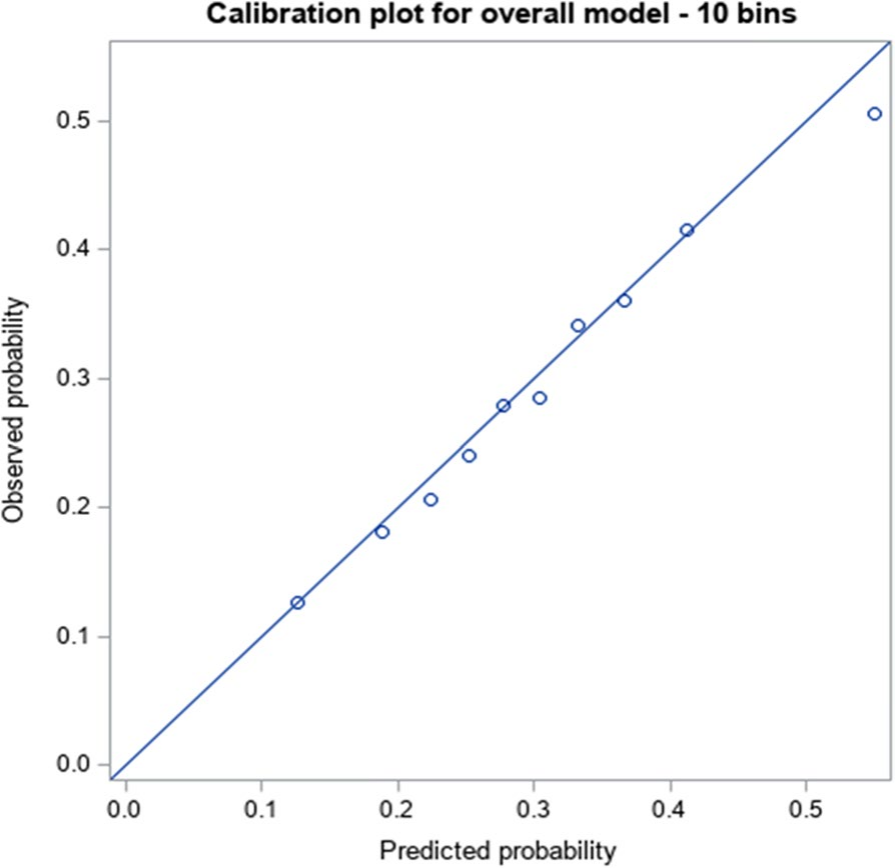
Calibration plot in the validation cohort. Dots represent each bin’s observed 6-month probability of CPS decline plotted against the 6-month predicted probability of CPS decline (among individuals in that bin)

When assessing performance of the risk prediction model using the validation cohort, we found that the discrimination was good, with an area under the receiver-operating characteristics curve of 0.6516 (Fig. 1) and calibration was excellent (Fig. 2). Calibration plots, among the various sub-groups, revealed only minor deviations from the overall results (data not shown), indicating that there was virtually no change in model performance based on any of the characteristics that were explored (e.g., among men and women, the c statistic was 0.65 in both cases).

Table 3 provides a comparison of baseline characteristics among individuals in the lowest and highest risk prediction deciles from the 10-bin calibration plot.

**Table 3:** Comparison individuals in the lowest and highest risk deciles from the 10-bin calibration plot under the validation cohort

	Bin 0 (lowest risk decile) *n* = 979	Bin 9 (highest risk decile) *n* = 979	*p*-value
	% (n)		
**Age (years)**			
18–64	79.8 (781)	1.5 (15)	< 0.0001
65–74	16.8 (164)	7.5 (73)	
75–84	3.2 (31)	35.1 (344)	
85 +	0.3 (3)	55.9 (547)	
**Sex**			
Male	46.4 (454)	48.3 (473)	0.39
Female	53.6 (525)	51.7 (506)	
**Marital status**			
Never married	22.1 (216)	3.5 (34)	< 0.0001
Married	52.2 (511)	29.1 (285)	
Widowed	10.0 (98)	40.5 (396)	
Separated/divorced	12.9 (126)	8.4 (82)	
Unknown/other	2.9 (28)	18.6 (182)	
**Baseline Cognitive Performance Scale (CPS) score**			
0	90.7 (888)	24.4 (239)	< 0.0001
1	9.3 (91)	75.6 (740)	
**Instrumental Activities of Daily Living (IADL) Involvement scale**			
None/mild difficulty (0–13)	86.3 (845)	48.2 (472)	< 0.0001
Moderate/major difficulty (14–21)	13.7 (134)	51.8 (507)	
**Pain scale**			
No pain/less than daily pain (0–2)	74.4 (728)	88.8 (869)	< 0.0001
Severe/daily pain (3–4)	25.6 (251)	11.2 (110)	
**Caregiver Risk Evaluation (CaRE)**^**1**^			
Low risk	23.6 (231)	21.3 (208)	< 0.0001
Moderate risk	39.7 (389)	29.6 (290)	
High risk	32.4 (317)	46.9 (459)	
Very high risk	n/a	n/a	
Missing	4.3 (42)	2.3 (22)	
**Coronary artery disease**			
No	85.5 (837)	80.5 (788)	0.0032
Yes	14.5 (142)	19.5 (191)	
**Alzheimer’s dementia**			
No	100.0 (979)	92.4 (905)	< 0.0001
Yes	0.0 (0)	7.6 (74)	
**Dementia (not Alzheimer’s dementia)**			
No	100.0 (979)	70.5 (690)	< 0.0001
Yes	0.0 (0)	29.5 (289)	
**Parkinson’s disease**			
No	100.0 (979)	82.6 (809)	< 0.0001
Yes	0.0 (0)	17.4 (170)	
**Any psychiatric diagnosis**			
No	88.4 (865)	79.9 (782)	< 0.0001
Yes	11.6 (114)	20.1 (197)	
**Sudden/new onset change in mental functioning (last 7 days)**			
No	99.9 (978)	97.3 (953)	< 0.0001
Yes	0.1 (1)	2.7 (26)	
**Stroke**			
No	94.3 (923)	81.0 (793)	< 0.0001
Yes	5.7 (56)	19.0 (186)	
**Hip fracture**			
No	96.9 (949)	97.1 (951)	0.7898
Yes	3.1 (30)	2.9 (28)	
**Other types of fracture**			
No	91.6 (897)	93.6 (916)	0.1011
Yes	8.4 (82)	6.4 (63)	
**Pneumonia**			
No	95.4 (934)	98.5 (964)	< 0.0001
Yes	4.6 (45)	1.5 (15)	
**Cancer**			
No	60.5 (592)	92.4 (905)	< 0.0001
Yes	39.5 (387)	7.6 (74)	
**Hearing impairment**			
No hearing impairment	94.4 (924)	67.8 (664)	< 0.0001
Hearing impairment	5.6 (55)	32.2 (315)	
**Vision impairment**			
No vision impairment	95.3 (933)	87.6 (858)	< 0.0001
Vision impairment	4.7 (46)	12.4 (121)	
**Dual sensory impairment**			
No dual sensory impairment	99.6 (975)	75.9 (743)	< 0.0001
Dual sensory impairment	0.4 (4)	24.1 (236)	
**Ability to understand others**			
Understands	99.0 (969)	69.9 (684)	< 0.0001
Usually understands	1.0 (10)	26.2 (256)	
Often understands	0.0 (0)	3.5 (34)	
Sometimes understands	0.0 (0)	0.3 (3)	
Rarely/never understands	0.0 (0)	0.2 (2)	
**Wandering**			
No	100.0 (979)	98.9 (968)	0.0009
Yes	0.0 (0)	1.1 (11)	
**Verbally abusive**			
No	99.0 (969)	95.5 (935)	< 0.00001
Yes	1.0 (10)	4.5 (44)	
**Chest pain**			
No	95.4 (934)	98.6 (965)	< 0.0001
Yes	4.6 (45)	1.4 (14)	
**Edema**			
No	71.7 (702)	80.6 (789)	< 0.0001
Yes	28.3 (277)	19.4 (190)	
**Hallucinations**			
No	100.0 (979)	96.5 (945)	< 0.0001
Yes	0.0 (0)	3.5 (34)	
**Clients believes that they are capable of increased functional independence**			
No	52.0 (509)	85.8 (840)	< 0.0001
Yes	48.0 (470)	14.2 (139)	
**Number of falls in last 90 days**			
0	88.5 (866)	47.7 (467)	< 0.0001
1	9.7 (95)	20.2 (198)	
2 +	1.8 (18)	32.1 (314)	
**Bladder incontinence**			
No	78.2 (766)	47.0 (460)	< 0.0001
Yes	21.8 (213)	53.0 (519)	
**Prognosis of less than six months to live**			
No	93.3 (913)	99.7 (976)	< 0.0001
Yes	6.7 (66)	0.3 (3)	
**Hospital admissions in the last 90 days**			
0	42.4 (415)	69.9 (684)	< 0.0001
1	47.9 (469)	24.3 (238)	
2 +	9.7 (95)	5.8 (57)	
**Client indicates that he/she feels lonely**			
No	91.6 (897)	75.3 (737)	< 0.0001
Yes	8.4 (82)	24.7 (242)	
**Change in social activities in the last 90 days**			
No decline/decline, not distressed	77.2 (756)	89.1 (872)	< 0.0001
Decline, distressed	22.8 (223)	10.9 (107)	

Compared to the lowest risk prediction bin, individuals in the highest bin were more likely to be 85 + years of age (55.9% vs. 0.3%), widowed (40.5% vs. 10.0%) and have a baseline CPS score of one (75.6% vs. 9.3%). Additionally, the highest bin was also more likely to experience moderate/major difficulty completing IADLs (51.8% vs. 13.7%) and have caregivers at high risk of experiencing caregiver burden based on the CaRE algorithm (46.9% vs. 32.4%). In terms of disease diagnoses, those in the highest risk group were more likely to have a diagnosis of Alzheimer’s dementia or another type of dementia, Parkinson’s disease, any psychiatric diagnoses, and stroke. Experiencing two or more falls (32.1% vs. 1.8%), having bladder incontinence (53.0% vs. 21.8%) and self-reported loneliness (24.7% vs. 8.4%) were all more likely in the highest bin.

Conversely, individuals in the highest bin were *less likely* to experience severe/daily pain (11.2% vs. 25.6%) and to have a cancer diagnosis (7.6% vs. 39.5%). Additionally, those in the highest bin were less likely to experience edema (19.4% vs. 28.3%), feel as though they were capable of increased independence (14.2% vs. 48.0%), have a prognosis of less than six months to live (0.3% vs. 6.7%) and be admitted to the hospital at least once in the last 90 days (24.3% vs. 47.9%; Table 3).

## Discussion

In this study, we developed and validated a novel prediction tool to mark decline on the CPS score that can be used for home care clients. By using a very large sample of clinical assessment data of home care clients from across Canada, we achieved good discrimination and excellent calibration, supporting the use of these data for risk prediction. While knowledge of the factors that increase risk are of interest, the strength of the nomogram is that it provides a unique risk score for an individual as a function of their own characteristics including age, various disease diagnoses, sensory and communication status deficits, as well as self-reported loneliness, and the risk of caregiver burden. To our knowledge the interRAI data have never been used to explore this particular outcome, although interRAI data have been used to create other prediction tools for those with cancer [18, 19].

There are a few features of our nomogram that are unique. For example, multiple prediction tools, or risk models, have been developed to understand the progression from mild CI to a dementia diagnosis [53–59], but we found no other studies that used a nomogram to predict the risk of cognitive performance decline. Given the breadth of the RAI-HC assessment, we were able to tap into multiple domain areas known to be associated with the risk of cognitive impairment or dementia. For example, in two recent reports from the Lancet Commission, 12 potentially modifiable risk factors were shown to account for roughly 40% of the risk of cognitive impairment or dementia [16, 60]. In our modelling, we were able to consider nine of these 12 risk factors, but were unable to look at traumatic brain injury, obesity, and air pollution, since they are not included on the RAI-HC assessment.

In our final model, individuals at highest risk for a decline on the CPS score were older, more likely to be widowed, to have difficulty completing IADLs independently, and to have care providers at high risk of experiencing caregiver burden. They were also more likely to have several diagnoses related to neurological and psychiatric conditions such as dementia. While some of these factors are non-modifiable (e.g., age and sex), some clearly are able to be addressed by the home care team. For example, caregiver burden is associated with the care recipient’s cognitive status [4, 5], and is a known risk factor for placement in a LTC facility or nursing home [9, 28, 61, 62]. Some recent studies suggest that supporting caregivers can lead to reduced or delayed LTC admissions [63, 64].

While the inclusion of variables related to a dementia diagnosis may seem counter-intuitive, we chose to keep them for a couple of reasons. For example, less than 5% of the sample (4.3%) had a dementia diagnosis. In addition, previous research reported that 12% of those with a CPS score of zero had a neurological diagnosis. The authors felt that this was because the assessor believed that the disease was present, but the person was not exhibiting symptoms, resulting in the low score on the CPS [32].

Our nomogram can support home care clinicians in this process as they engage in shared decision-making with the person and their family. However, like other health index scales and algorithms embedded within the interRAI tools, clinicians should use the information from this new prediction tool to augment, but not replace, their clinical judgement. Home care clinicians would ideally use this information, in conjunction with conversations with the person and their family, to ensure that decisions are tailored to best meet their needs.

Our risk prediction model utilized data from five Canadian provinces and one territory. This large cohort represented adult (aged 18 +) home care clients with a variety of illnesses and symptoms and in various stages of their illness trajectory. As a result, it may be generalizable to other publicly-funded home care systems. Although we found no other prediction tools for a decline on the CPS, several studies have created prediction tools for predicting a diagnosis of dementia. For example, two studies reported the c statistics across data analyzed from several existing cohort studies, ranging from 0.64 to 0.78 [57, 58]. The lower values in these studies are in line with our level of discrimination (c = 0.65). However, our model was not as good as two other studies, both of which used existing administrative or survey data, and had values of 0.74 [55] and 0.82 [54]. This likely reflects the added difficulty and complexity of predicting the risk of deterioration on the CPS score versus predicting a dementia diagnosis.

One limitation to our work is that the RAI-HC assessment included no information about biologic markers such as imaging data and serologic findings, which can be important in determining the risk of cognitive impairment or dementia [56, 59]. Since we explored changes on the CPS, individuals with a single assessment were necessarily excluded from the cohort. However, it is unclear how this may have biased the sample since several scenarios would be operating simultaneously within the six-month timeframe. For example, some home care clients would have a single assessment because they died or were admitted to a LTC facility. Others, however, would only have one assessment since they improved and were subsequently discharged from the home care program. Furthermore, the CPS is a functional screening tool and cannot be used for diagnostic purposes, although it has been noted that individuals with higher scores (i.e., three or higher on the CPS) are more likely to have a diagnosis of Alzheimer’s dementia versus those scoring two or lower [40].

## Conclusions

Our model demonstrated that the risk of a decline on the CPS score can be accurately predicted using existing data from the RAI-HC. Using data from multiple domain areas showed the prediction tool tap into risks related not only to neurologic conditions, such as Alzheimer’s or a related dementia, but also the risks associated with sensory impairments, caregiver burden, and communication status. As a result, the tool possesses strong potential to provide clinicians with unique risk information for a given person. It can be used by them to guide further assessment, referrals, and help to support their clinical decision-making as they work with clients and families to navigate the health care system. Since the interRAI tools are widely used around the world, there is strong potential for this new nomogram to be utilized to generate information for use by home care professionals in multiple countries.

## Data Availability

The dataset analyzed during the current study is not publicly available since the interRAI assessments are shared by various provinces and territories with limited access in the respective data sharing agreements. The data can be requested from the Canadian Institute for Health Information (https://www.cihi.ca/en/access-data-and-reports/make-a-data-request).
